# An Unusual Case of a Perforated Meckel's Diverticulum

**DOI:** 10.1155/2023/2289520

**Published:** 2023-04-21

**Authors:** Lybil Mendoza Alvarez, Dhanashree Rajderkar, Genie L. Beasley

**Affiliations:** ^1^University of Tennessee Health Science Center Pediatric Gastroenterology, Memphis, TN, USA; ^2^University of Florida Pediatric Radiology, Gainesville, FL, USA; ^3^University of Florida Pediatric Gastroenterology, Gainesville, FL, USA

## Abstract

**Background:**

Meckel's diverticulum, the most common congenital anomaly of the gastrointestinal tract, typically presents in children with gastrointestinal bleeding. *Case Presentation*. An 11-year-old Caucasian male presented with a 6 week history of abdominal pain, vomiting, and diarrhea. He was found to have iron deficiency anemia, markedly elevated serum and fecal inflammatory markers, and imaging showing a contained bowel perforation. He was evaluated for infectious etiologies and later underwent extensive testing for inflammatory bowel disease. Ultimately, he was found to have a Meckel's diverticulum, which was successfully resected and led to resolution of his gastrointestinal complaints.

**Conclusions:**

This case report highlights one of the more rare presentations in children, which is intestinal perforation. Symptoms of a Meckel's diverticulum can overlap with those of inflammatory bowel disease, as demonstrated by our patient. Clinicians should be familiar with criteria to establish diagnosis of inflammatory bowel disease, and if diagnosis isn't fully supported by testing, they should expand the differential and consider Meckel's diverticulum.

## 1. Introduction

Meckel's diverticulum is a congenital diverticulum on the ileum caused by incomplete atrophy of the omphalomesenteric duct [1]. It is the most common congenital anomaly of the gastrointestinal tract. Meckel's diverticulum occurs in 2% of the population, and although most are clinically silent, those who become symptomatic often do so before 2 years of age. Based on one of the largest databases for children with symptomatic Meckel's diverticulum, only 2% of children become symptomatic. Of those with symptoms, 60% of children present with obstruction, 35% with gastrointestinal bleeding, and 8% with inflammation [1, 2]. Chronic iron-deficiency anemia has also been reported as an early manifestation of Meckel's diverticulum [3]. Painless gastrointestinal hemorrhage is the consequence of acid originating from a patch of ectopic gastric tissue in the Meckel's, which causes peptic ulcer formation within the diverticulum or disruption of the downstream intestinal mucosa. Inflammation occurs in the diverticulum itself, and perforation, a rare complication, can occur secondary to diverticulitis or ulcer within the Meckel's [1, 4].

Inflammatory bowel disease (IBD) is a chronic immune-mediated condition of the gastrointestinal tract, with a rising incidence within the last 10 years. Approximately 25% of patients with IBD are diagnosed before twenty years of age [5]. IBD can present with a variety of symptoms such as abdominal pain, diarrhea, rectal bleeding, weightloss, or growth failure. Patients can also present with surgical emergencies including peritonitis, small bowel obstruction, appendicitis, or abscess. If an otherwise healthy child presents with an acute intestinal surgical emergency accompanied by poor growth and signs of chronicity, there is always a concern for inflammatory bowel disease, primarily Crohn's disease [5].

The authors present a case of an 11-year-old male with initial clinical picture of IBD, subsequently found to have perforated Meckel's diverticulum with abscess formation. This case is unique because it is one of just a few cases of a perforated Meckel's in children. Through this case, clinicians can learn about this rare complication of Meckel's, and how many symptoms and findings on the clinical work-up can overlap with Crohn's disease.

## 2. Case Presentation

**Figure 1:**
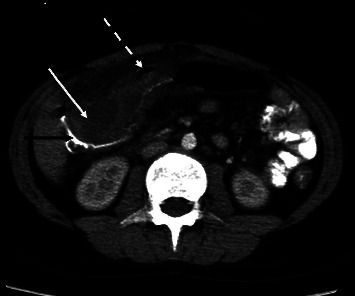
CT of abdomen and pelvis with intravenous and oral contrast showed severe wall thickening (black arrow) of the ascending colon with adjoining phlegmon (white arrow), and small amount of adjacent free air suggesting contained perforation (dotted white arrow).

An 11-year-old male with no past medical history, travel history, or family medical history presented with a 6-week history of gradually worsening periumbilical pain and two weeks of nonbilious vomiting, nonbloody diarrhea, and fever. History was negative for extra-intestinal symptoms such as joint pain or swelling, oral ulcers, eye pain, or skin rash. His physical exam was significant for diffuse abdominal tenderness but guarding or distention. Computerized tomography (CT) scan of abdomen and pelvis showed signs of inflammation in the ascending and transverse colon, and a contained bowel perforation (Figure 1). Laboratory investigation was significant for white blood cell count of 6 thou/cu mm (normal 4.5–13.5 thou/cu mm), hemoglobin of 9.9 g/dL (normal 11.5–15.5 g/dL), C-reactive protein of 221 mg/L (normal 0–5 mg/L), albumin of 2.5 g/dL (normal 3.5–5.2 g/dL), ferritin of 9 ng/mL (normal 24–336 ng/mL), and negative PCR stool studies for viral, bacterial, and parasitic infection. Fecal calprotectin was significantly elevated, at >1250 *μ*g/g (normal 0–50 *μ*g/g). We suspected a diagnosis of IBD versus a prior severe infectious colitis that was not detectable by stool PCR testing. Because the patient did not have free air in the abdomen or peritoneal signs, laparotomy was deferred and the patient received 14 days of broad-spectrum antibiotics, which lead to resolution of abdominal pain, vomiting, diarrhea, and fever.

After completing antibiotics, esophagogastroduodenoscopy (EGD) and colonoscopy revealed grossly normal intestinal mucosa, with biopsy findings of focal active ileitis and colitis, nonspecific and insufficient for diagnosis of IBD. Intestinal tuberculosis was considered, but QuantiFERON-TB test was negative, and imaging and histology did not support this diagnosis. 4th-generation IBD diagnostic test (commercial in US PROMETHEUS) which combines serologic, genetic, and inflammation markers including 9 serological markers: ASCA IgA, ASCA IgG, and proprietary markers anti-Fla-X, anti-A4-Fla2, anti-CBir1, anti-OMPC, and DNAse-sensitive pANCA that helps identify patients with IBD was negative for IBD, and repeat fecal calprotectin, serum inflammatory markers, complete blood count, and albumin had all normalized, without any treatment for IBD. The patient then developed daily bloody stools and was started on infliximab based on symptoms and ileal inflammation on MRE, though need for further testing with wireless capsule endoscopy was being planned to further solidify diagnosis. Several weeks after initiating treatment for IBD, the patient presented with worsening of abdominal pain and fever. A CT scan revealed signs suggestive of a 7.6 × 4.3 × 7 cm phlegmon adjacent to the bowel in the right lower abdomen.

**Figure 3:**
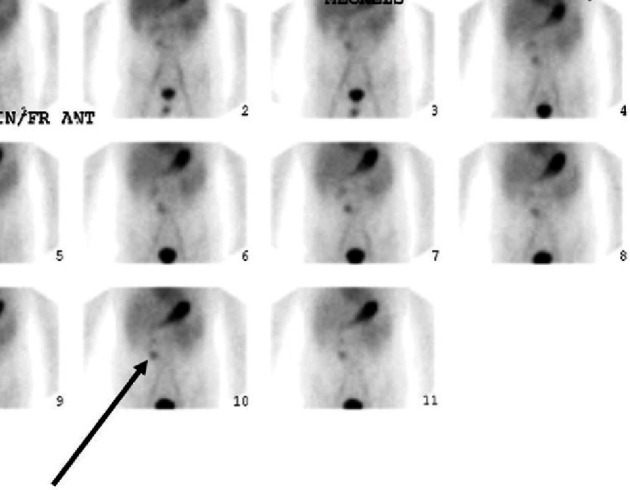
A technetium 99 m pertechnetate scan showed increased focal uptake in the right lower quadrant consistent with Meckel's diverticulum with ectopic stomach mucosa (black arrow).

With work-up not definitive for IBD, other causes for intra-abdominal abscess were considered, and concern for Meckel's diverticulum was raised. A technetium 99 m scan was obtained, and findings were consistent with Meckel's diverticulum (Figure 3). Nine months after this patient's onset of symptoms, laparoscopic exploration was performed, and a Meckel's diverticulum was identified. At the time of surgery, there was no abscess and no sign of perforation. The diverticulum was covered by a rind of inflammatory tissue, and the location of this inflammatory mass was concordant with the location of the phlegmon on the aforementioned CT scan. Another finding at the time of surgery was the presence of adhesions. Resection diverticulectomy with side-to-side anastomosis was performed. Histology confirmed this diagnosis. Patient's abdominal pain and hematochezia resolved after surgery. The patient was followed for 15 months after surgery and has remained asymptomatic since then.

## 3. Discussion

A Meckel's diverticulum simulates the shape of a duplication cyst or blind pouch separated from the ileum when observed on abdominal ultrasound and CT. Meckel's might be difficult to distinguish from the adjoining small bowel loops on imaging, making diagnosis by these imaging modalities more challenging. Pertechnetate scintigraphy, with H2 receptor blocker given before the scan to improve accuracy, provides a diagnostic sensitivity of 94%–100% and a specificity of up to 97–100% in diagnosing heterotopic gastric mucosa within a Meckel's diverticulum in children although it is operator dependent [6]. The treatment of choice is surgical resection [2].

Our case is that of a child with subacute nonbloody diarrhea and initial radiologic findings of severe infectious colitis versus inflammatory bowel disease in the right hemi-abdomen, with contained perforation. Such findings plus clinical symptoms and initial laboratory evaluation (iron deficiency with anemia, hypoalbuminemia, and elevated serum and fecal inflammatory markers) made us suspect a diagnosis of IBD initially, since up to 28% of patients with newly diagnosed CD can present with penetrating (intra-abdominal abscesses or fistula) or structuring disease; however, the incidence of intra-abdominal abscesses alone is not known [5]. Though biopsy results with minimal focal active ileitis and colitis were nonspecific for IBD, we entertained the possibility that he could have early evolving IBD. The patient's MRE raised suspicion for small bowel CD, and treatment for IBD was initiated based on overall clinical picture.

Cases of perforated Meckel's with subsequent abscess formation have been found in adults [7], but there are few in children [3, 4]. These rare cases of perforation made us suspect a Meckel's diverticulum in our patient [4]. Our particular case is unique because it is one of few case reports of a child with chronic gastrointestinal symptoms (ultimately, he had 9 months of abdominal pain with nonbloody diarrhea), laboratory findings, histology, and imaging showing bowel perforation, raising suspicion for a diagnosis of IBD, but later found to have Meckel's instead. Our patient's iron deficiency, anemia, and hypoalbuminemia at initial presentation were likely a result of chronic, unrecognized slow gastrointestinal bleeding. Although there are some case reports of Meckel's diverticula found in patients with Crohn's disease [8], a subsequent study of 877 patients with CD found no increased incidence of Meckel's in patients with CD [9].

Clinicians should consider Meckel's diverticulum as a potential cause of surgical abdomen such as abdominal abscess or intestinal perforation. Additionally, when a patient presents with clinical features of IBD but histology does not support diagnosis, or patient is not responding to reasonable therapy, other diagnoses which may have overlapping presentation should be considered. Clinicians should certainly have a broad differential on initial assessment when patients (children any age) present with symptoms of abdominal pain, blood in stools, diarrhea, fever, anemia, elevated inflammatory markers, and/or findings of inflammatory bowel disease in imaging such as CT abdomen/pelvis. Once patient undergoes esophagogastroduodenoscopy/colonoscopy with biopsies that are not convincing for inflammatory bowel disease as well as poor response to conventional therapies for inflammatory bowel disease. Patient should be followed very closely mostly if symptoms of bloody stools consider performing a Meckel's scan to look for Meckel's diverticulum.

## Figures and Tables

**Figure 2:**
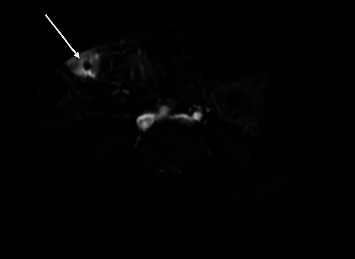
Magnetic resonance enterography performed with intravenous contrast showed short segment of ileal inflammation with wall thickening and enhancement (white arrow).

## Data Availability

The data supporting the current study are available from the corresponding author upon request.

## Abbreviations

IBD: Inflammatory bowel disease
CT scan: Computerized tomography
MRE: Magnetic resonance enterography
CD: Crohn's disease.

## Additional Points

First author: Lybil Mendoza Alvarez institutional address: lmendoz2@uthsc.edu.

## References

[1] Hansen C. C., Søreide K. (2018). Systematic review of epidemiology, presentation, and management of Meckel’s diverticulum in the 21st century. *Medicine (Baltimore)*.

[2] Rattan K., Singh J., Dalal P., Rattan A. (2016). Meckel’s diverticulum in children: our 12-year experience. *African Journal of Paediatric Surgery*.

[3] Kang H. S., Lee J. S., Hyun C. R., Jung I. H., Kang K. S. (2019). Meckel’s diverticulum diagnosed in a child with suspected small bowel crohn’s disease. *Pediatr Gastroenterol Hepatol Nutr*.

[4] Keese D., Rolle U., Gfroerer S., Fiegel H. (2019). Symptomatic meckel’s diverticulum in pediatric patients—case reports and systematic review of the literature. *Front Pediatr*.

[5] Conrad M. A., Rosh J. R. (2017). Pediatric inflammatory bowel disease. *Pediatric Clinics of North America*.

[6] Irvine I., Doherty A., Hayes R. (2017). Bleeding meckel’s diverticulum: a study of the accuracy of pertechnetate scintigraphy as a diagnostic tool. *European Journal of Radiology*.

[7] Hong J., Park S. B. (2017). A case of retroperitoneal abscess: a rare complication of Meckel’s diverticulum. *International Journal of Surgery Case Reports*.

[8] Andreyev H. J., Owen R. A., Thompson I., Forbes A. (1994). Association between Meckel’s diverticulum and Crohn’s disease: a retrospective review. *Gut*.

[9] Freeman H. J. (2001). Meckel’s diverticulum in Crohn’s disease. *Canadian Journal of Gastroenterology*.

